# Validation of the Patient-Reported Outcome Measures Tool “Catquest” in Odia Language

**DOI:** 10.7759/cureus.82071

**Published:** 2025-04-11

**Authors:** Saswati Sen, Matuli Das, Kavitha AK, Aiswariya Mohanty, Anjali Goel

**Affiliations:** 1 Ophthalmology, Kalinga Institute of Medical Sciences, Bhubaneswar, IND; 2 Public Health, Indian Council of Medical Research (ICMR), Regional Medical Research Centre, Bhubaneswar, IND

**Keywords:** cataract, catquest, patient-reported outcome measures, reliability, validity

## Abstract

Background: Healthcare is evolving to consider patient satisfaction along with any procedure’s objective outcome. After cataract surgery, the quality of vision can be assessed by administering questionnaires on daily activities. Using such questionnaires in local languages enhances patient-doctor interaction. In this study, we aim to determine whether using a Catquest questionnaire in Odia can help better assess the quality of life in the setting of a tertiary care center in Eastern India.

Objectives: This study aims to validate the use of a Catquest questionnaire among Eastern India’s Odia-speaking population.

Methodology: We conducted a prospective study on 40 patients planning to undergo cataract surgery in a tertiary care center in Eastern India. We collected demographic data and conducted a comprehensive ocular examination, including slit lamp examination, fundus examination, and intraocular pressure (IOP) measurement. We asked patients to complete a Catquest questionnaire before and six weeks after surgery. We translated the English version of the questionnaire into Odia and validated it for use in the study. Patients completed both Odia and English questionnaires on both occasions. We conducted a statistical analysis to check for agreement between the answers in both languages and to validate the questionnaire in Odia. First translation and back-translation were done in English and Odia. We prepared a final draft of the questionnaire in Odia. We determined intraclass correlation coefficient (ICC) values and compared the reliability of the responses of the same group of patients before and after surgery. We explored variability between answers in both languages.

Results: The data were categorical. ICC values for various activities such as reading papers, seeing to walk on uneven ground, watching television, and preferred hobbies showed perfect reliability, with ICC = 1 both preop and postop. This indicated consistent perceptions of these activities across the two time points. Activities such as seeing prices, seeing to do needlework, reading newspapers, and shopping also showed good reliability. For reading text on television, the ICC values were -0.0588 preop. However, postop, all patients were able to watch television comfortably. Thus, there was no variability between the answers. Negative ICC preop indicated very poor reliability or inconsistency preop, which we corrected postop.

Conclusion: With the growing emphasis on patient-reported outcomes, both clinical and subjective parameters need to be considered for any procedure’s success. In our clinical setting, the use of the Catquest questionnaire for assessing patient-reported outcomes in their day-to-day activities was beneficial. The translated version was reliable and well-applicable in the setting of the present study. As the study shows a good correlation between the original and translated versions, we can conclude that it can be incorporated in clinical practice successfully.

## Introduction

Cataracts are the most common cause of avoidable blindness worldwide [1]. The surgical treatment for cataracts has greatly improved to offer better results both objectively and subjectively. Postsurgery, objective improvement can be documented by assessing visual acuity (VA), intraocular pressure (IOP), or residual refractive error in addition to the clinical assessment of the anterior and posterior segments of the eye. Subjective assessment or satisfaction postsurgery includes tools such as robust questionnaires that may effectively report patients’ perceptions before and after surgery [2]. Based on this background, several such tools have been devised and modified over time to assess patient perceptions [3,4]. These tools form the basis of the present study. The Catquest questionnaire is one such questionnaire designed to assess the patient-reported outcome measures related to cataract surgeries [5]. The success of surgery in the current scenario not only depends on postoperative VA but also on the ability of the patient to carry out certain activities postsurgery. It also depends on whether the patient is able to overcome certain perceived problems or disabilities that they faced prior to the surgery. The Catquest questionnaire has a set of questions that attempt to assess subjective improvement by asking questions on perceived disabilities that may affect one or more vision-related functions. It is a closed-ended questionnaire, which enables better evaluation of the level of subjective improvement in our patients. First, translating and then validating the questionnaire helps patients who speak the local language understand the questions more easily. This, in turn, will provide a clearer perception of patient satisfaction as well as enhance the surgeon’s understanding of the problems patients face on a day-to-day basis [6]. The present study aims to use this questionnaire in the local language and determine whether it is relevant in assessing patient-reported outcomes in the developing world. The rationale is to use the preset questionnaires in our setup. This precludes the need to frame new questions and allows a better comparison among studies using the same questionnaire. The present study was previously posted on the medRxiv preprint server in November 2024 and is not under review in any other journal for publication.

## Materials and methods

Study design

This was a prospective study conducted in a tertiary care hospital in eastern India (Odisha).

Ethical considerations

After obtaining the Institutional Ethical Committee clearance (KIIT/KIMS/IEC/952 ), the study was conducted according to the principles of the Declaration of Helsinki. The study period was from June 2023 to December 2023.

Sampling, study procedure, and tools

Purposive sampling was used for the study. A total of 40 patients were included in the study. These were patients reporting to the ophthalmology outpatient department with operable cataract who underwent evaluation and surgery for cataract in the department of ophthalmology of a tertiary care center in Eastern Odisha. Eligible patients were identified to be included in the study, and written informed consent was obtained. Demographic profile of all the patients was recorded, and a thorough clinical history and examination on slit lamp was done. All patients included in the study were bilingual. They could read, write, and understand both Odia and English without any assistance. The decision on the type of surgery was based on the grade and type of cataract.

Patients older than 40 years and having cataracts with a nuclear sclerotic cataract (NS) grade of 2 and above as per the Lens Opacities Classification System, Version III (LOCS III) grading were included in the study. We excluded patients who refused to participate in the study; patients having any eye disease causing profound vision loss other than brown cataracts, such as optic nerve damage or retinal diseases; or any patient having preexisting systemic or ocular disease causing visual discomfort or visual loss other than cataracts. All eyes of patients in the study underwent a thorough ophthalmic examination as preoperative requisites for cataract surgery. The examination included VA testing using the Snellen chart and the log medication administration record (MAR) chart, slit lamp biomicroscopy for anterior segment examination, and color vision and contrast sensitivity. We conducted dilated fundoscopic examinations with a +90 D lens and slit lamp biomicroscopy. We also used a +20 D lens and indirect ophthalmoscopy of the posterior segment to rule out any other causes of visual defects, such as retinopathies or retinal detachment. IOP was measured using Goldmann Applanation Tonometry. Catquest questionnaires in both English and Odia were administered to the patients, both before and six weeks after the surgery. Data from all the completed forms were collected and analyzed. The best possible correction in the form of glasses was given to all before collecting responses.

Process of translation and validation

The validated English version of the Catquest questionnaire, available in the public domain, was first translated into Odia by two independent translators (Appendix 1). Both translators were bilingual and could read and write in both English and Odia. After careful discussion and consideration, we reached a consensus on a single version to be used in the study. The panel decided to use simple Odia terms that would be understandable to the layman to make it easier for patients to understand the nature of the questions. The Odia version was also reviewed by several monolingual members, and we took their suggestions into account before further modification of the questionnaire. This Odia version was then again back-translated into English. The English version of the validated Catquest questionnaire and the back-translated English version were again compared by our experts. A few words and phrases in Odia were changed or modified. Some other words, like *suikama*, hobby, embroidery, and headlight, were used as they appeared, but we did not observe much discrepancy in the patients’ understanding of the essence of the questions. After confirming that the meaning and language had not been altered, we developed a final Odia version, which was approved by the expert committee. This Odia version was then used in the study (Appendix 2).

Statistical analysis

The data collected were categorical in nature. Hence, the intraclass correlation coefficient (ICC) values were used to find the reliability and validity of the translated items in the questionnaire. The ICC values were calculated using a one-way random-effects model with the agreement type to assess the consistency of measurements across different time points. ICC measures the degree of consistency or reliability of measurements across different time points or conditions, with values ranging from 0 to 1, with potentially negative values indicating very poor reliability. The tables in the study were then framed to derive ICC values for various activities and perceived problems both before (preop) and after (postop) the operation.

## Results

A total of 40 patients were included in the study. The majority of patients were more than 60 years old. There was no significant difference in gender, with an equal number of males (20) and females (20) being operated on for cataracts. More males (19) underwent phacoemulsification, and 15 females underwent small-incision cataract surgery (SICS). When compared, the majority of patients had an educational level of postgraduate or higher. In addition, more patients were above the poverty line (25 (62.5%)) than below the poverty line (15 (37.5%)). With respect to gender, there was no statistical difference between these demographic parameters. This meant that patients from both genders had similar educational and financial status. But when it came to surgical procedures, there was a significant difference in the surgical procedures conducted on each gender. The details are listed in Table 1.

**Table 1:** Descriptive summary of study participants SICS: small-incision cataract surgery Chi-square test was applied, and a p-value of <0.05 was considered to be significant

Variables		Male n (%)	Female n (%)	Total (N = 40)	p-value	Chi-square
Age	<60 years	2 (10%)	5 (25%)	7 (17.5%)	0.211	1.558
	>60 years	18 (90%)	15 (75%)	33 (82.5%)		
Education	Graduate (12th pass)	9 (45%)	6 (30%)	15 (37.5%)	0.327	0.96
	Postgraduate	11 (55%)	14 (70%)	25 (62.5%)		
Income	Above poverty line	11 (55%)	14 (70%)	25 (62.5%)	0.327	0.96
	Below poverty line	9 (45%)	6 (30%)	15(37.5%)		
Surgical procedure	Phaco	19 (95%)	5 (25%)	20 (50%)	<0.001	20.416
	SICS	1 (5%)	15 (75%)	16 (40%)		
Total		20 (50%)	20 (50%)	40 (100%)		

The questionnaire had three subsections and 18 items in total, with room for multidimensional answers. Here, lower numbers (1) represented a lower degree of disability, and higher figures represented a higher degree of disability. The questions represented disability based on cataract symptoms, day-to-day activities, and preferred hobbies that were affected by the disease. Although the answers to each question were evaluated separately, seven items were broadly categorized and evaluated. The questionnaire was administered to the patients before and then again four weeks after the surgical procedure. ICC was calculated for the responses given in the English and Odia questionnaires because the data were categorical. These values represented the reliability of the responses given for the same questions in English and Odia (Table 2).

**Table 2:** Corelation between Odia and English questionnaire comparing day-to-day activities Intraclass correlation coefficient (ICC) was used for testing, and a p-value of <0.05 was considered significant

Items	Preop		Postop	
	Intraclass correlation coefficient (ICC)	p-value	Intraclass correlation coefficient (ICC)	p-value
Reading papers	1.00	<0.01	1.00	<0.01
Recognizing faces	1.00	<0.01	NA	-
Seeing prices/shopping	0.814	<0.01	1.00	<0.01
Seeing to walk on uneven ground	1.00	<0.01	1.00	<0.01
Seeing to do needlework	0.88	<0.01	1.00	<0.01
Reading text on television	0.21	<0.04	0.21	<0.04
Seeing to perform a preferred activity	0.87	<0.01	1	<0.01

Higher values represented better reliability and validity. For items like reading papers, recognizing faces, and seeing to walk on uneven ground, the ICC was 1 preoperatively, which showed good patient understanding of questions pertaining to these activities in both languages. Postoperatively, except for recognizing faces, the other two categories showed good ICCs. Because of a significant improvement in their vision, the patients were able to recognize faces without any problem. Perhaps that was why the question did not seem more relevant postoperatively in a broad sense. Maybe questions about finer VA assessment would have judged the parameter more accurately in our setting. Other activities like reading prices on shopping items, doing needlework, or engaging in a preferred hobby showed good ICCs both preoperatively and postoperatively in both languages, indicating that the questions were interpreted similarly in both languages. The ICC for doing needlework was 0.88 preop and 1 postop, indicating a mild change in reliability or consistency in the perception of problems related to this activity, with minimal improvement observed after the operation.

The only category that showed lower ICC values was reading text on television. Our analysis indicated that some terms used in the Odia questionnaire were confusing for the patients. The terms *Dooradarshana* and TV were used interchangeably. After we made some changes, the responses improved. Another reason for low values was that many patients were not prescribed correction in the form of glasses after surgery, which may have led to them skipping the question altogether or giving erroneous responses. The ICC postop was not derivable; hence, not applicable (NA) was mentioned in the table. This is attributed to the fact that no patients reported difficulty with reading text on television after the operation, resulting in no variability to measure agreement against. The responses were similar in both languages. Overall, the results indicated good agreement between the questionnaires in both languages and showed that both were understandable to the local population.

## Discussion

Patient satisfaction is paramount after every surgery. In ophthalmology, even after a simple cataract surgery resulting in excellent vision and other objective parameters, patients may still not be satisfied [7]. With changing treatment perspectives, the use of questionnaires has become a desirable way in which to assess the subjective parameters, and the Catquest questionnaire has been used extensively for such assessment in various countries [8]. In addition to Catquest, several other questionnaires have been used similarly [9,10]. Although the questions per se may be different, the overall aim and structure are meant to evaluate visual disability and assess the patient-reported outcomes pre- and postsurgery.

The aim of the present study is to validate the Catquest questionnaire in the local language so that patient perspectives are easily understandable on their own terms. This included trying to use as many local terms as possible in day-to-day practice so that patients could more easily understand the questions and how to express the answers. Translation improves communication with patients and increases the reach among the study population. Communication in the local language also builds trust between the healthcare professionals and patients [11]. Further validation of this paper ensures that the content is correct as far as the context is concerned. This means that patient problems can be correctly identified and not “lost in translation” [12,13].

The questions used on the Catquest questionnaire are closed-ended questions divided into three sections: daily activities, difficulty in performing those activities, and questions on general health. The questionnaire itself and its modified form have been translated and validated in several languages [14-17]. These questions can be extrapolated to our sociodemographic scenario as well, which will provide insight into the patient’s perspective. Hence, the first step was to translate the questions into the vernacular. This process included the use of colloquial terms rather than formal language, which made understanding the questionnaire easier. Although initially the volunteers did help the patients understand the process of answering the questions, by the end, care was taken to record the patient’s interpretation [18,19].

The subjects in the present study did not have any significant socioeconomic disparities except for the choice of surgery in the cases of males and females. This may be explained by the fact that phacoemulsification in brown cataracts warrants more caution than SICS, and males may be more predisposed to take risks than females in our society [20]. But the Catquest questionnaire comprised questions related to day-to-day activities, which were basically gender-neutral. Hence, answers were not biased by the patients’ lifestyle.

Questions about activities like needlework, reading prices on items, and reading newspapers were well understood and interpreted in both languages. Questions pertaining to watching television showed no variability postop. This was probably due to a problem in interpreting the words used, which were then modified and later confirmed to be understandable by the study subjects.

Responses to questions may also be guided by the fact that people who perform some activities more often may be less likely to report difficulties in performing the task because of greater adaptability [21]. A previous study using Catquest with an Indian population needed to alter some questions to cater to the Indian context [22]. But the present study showed good correlation and agreement for the questions in the Odia language. English is not yet used as a first language in many parts of the world. Translation and validation of these questionnaires may improve doctor-patient communication because they combine both clinical and subjective aspects of treatment. This ensures improved empathy and helps in keeping up with the current trends and better patient care [23,24]. 

Our study is novel because it uses the Catquest questionnaire in the Odia language to assess patient-reported outcomes in cataract surgery, which has not been done before. This will help improve patient communication and counselling, although a bigger sample size could have validated the results in a better way.

## Conclusions

In the present study, we successfully translated the Catquest questionnaire into the Odia language. This combined both clinical objectives and subjective parameters to address patient expectations and concerns. The translation successfully addressed the hope that the questions were adaptable to our clinical setting and that using the questionnaire could help us better understand patient perspectives. The good reliability of the answers in both languages means that the Odia version worked well in the Odia-speaking cataract population. Hence, the validated Odia questionnaire can be incorporated into our clinical practice to ensure better patient care.

## Figures and Tables

**Figure 1:**
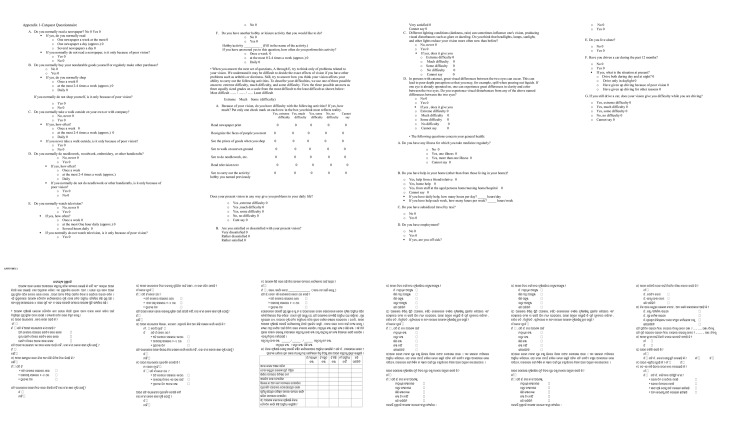
Appendix 1, Catquest questionnaire in English; Appendix 2, Catquest questionnaire in Odia
